# N-acetylcysteine for managing neurotic excoriation: encouraging results in two patients^☆^^☆☆^

**DOI:** 10.1016/j.abd.2020.06.021

**Published:** 2021-03-20

**Authors:** Deren Özcan

**Affiliations:** Department of Dermatology, Faculty of Medicine, Başkent University, Ankara, Turkey

Dear Editor,

Neurotic Excoriation (NE) is characterized by recurrent picking of skin, leading to cutaneous lesions ranging from superficial erosions to deep ulcerations.^1^ Acne excoriée (AE) is a subset of NE in which the focus is on acne lesions. NE causes significant psychosocial impairment, which therefore necessitates an effective treatment.^1^, ^2^ Although several approaches exist, including behavioral and pharmacological therapies, managing NE is still challenging.^1^, ^2^, ^3^ Glutamatergic dysfunction and oxidative stress are thought to contribute to the pathophysiology of NE.^2^, ^3^ Recently, N-Acetylcysteine (NAC), a glutamate modulator and an antioxidant, has been proposed as a promising treatment alternative for NE, and a limited number of reports have shown encouraging results.^1^, ^2^, ^3^, ^4^, ^5^

A 75-year-old woman presented with a 5-year history of itching and scattered wounds on her legs. She had been feeling an irresistible urge to pick her skin, which then became a daily routine that ensued self-inflicted lesions on otherwise normal-appearing skin. Dermatologic examination revealed multiple irregularly shaped erythematous or hyperpigmented, eroded, excoriated, or lichenified papules and nodules of varying size on both legs (Fig. 1A). Complete blood cell count and liver, renal and thyroid function test results were within normal limits. The second case was a 36-year-old woman who presented with a 3-month history of itchy acne-like lesions on her face. She had used many dermocosmetic products without success. She had been habitually picking, scratching, and squeezing these lesions, and despite these repeated efforts, she was unable to resist this behavior. Dermatologic examination revealed a few comedones, erythematous and excoriated papules, as well as hyperpigmented macules on the forehead (Fig. 2A).

**Figure 1:**
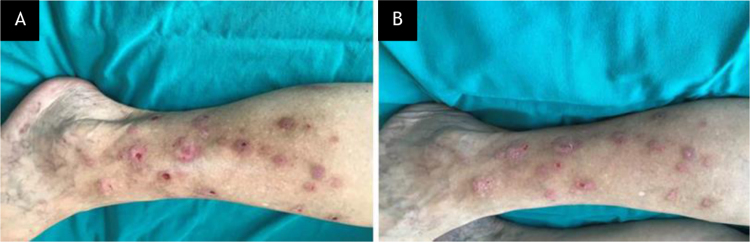
Case 1, (A), Multiple irregularly shaped erythematous or hyperpigmented, eroded, excoriated, or lichenified papules and nodules of varying size on the right leg of the patient with neurotic excoriation. (B), Partial clinical improvement after 2-weeks on N-acetylcysteine treatment.

**Figure 2:**
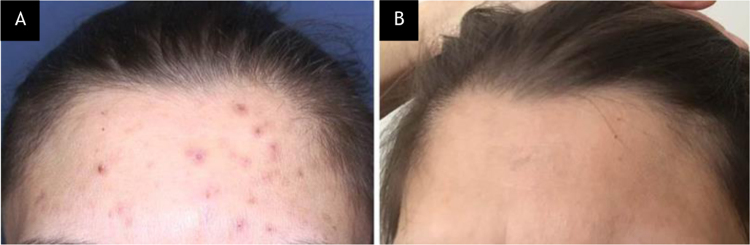
Case 2, (A), Erythematous and excoriated papules, and hyperpigmented macules on the forehead of the patient with acne excoriée. (B), Complete clinical improvement after 6-weeks on N-acetylcysteine treatment.

Tzanck smears taken from the lesions stained with May-Grünwald-Giemsa showed no pathology. Upon the diagnosis of NE and AE, respectively, both patients were started on NAC (1200 mg/d, p.o.). The clinical findings subsided after 2- and 6-weeks, in the first (Fig. 1B) and second (Fig. 2B) patient, respectively. No side effects were observed and both patients no longer displayed self-excoriation behavior. The treatment lasted 3-months and 6-weeks, and after cessation of therapy no relapse was observed in 6- and 3-months follow-up in the NE and AE patient, respectively.

There are only a few case reports and two studies in the literature that indicate the potential benefit of NAC for treating NE.^1^, ^2^, ^3^, ^4^, ^5^ In those reports, NAC dosage and treatment duration varied greatly (450–3000 mg/d and 1–10 months).^2^, ^3^ Side effects including gastrointestinal upset, dry mouth, and dizziness were rarely observed and did not require cessation of therapy.^2^, ^3^, ^4^, ^5^ Nevertheless, the follow-up data regarding the relapse risk after discontinuation of NAC are unknown.

NE is a psychocutaneous disorder, and given that those patients usually present to dermatology clinics, not only the psychiatrists but also the dermatologists should be aware of new treatment options. Our experience in the present two cases supports the notion that NAC could be a safe and effective alternative for managing NE. However, the appropriate NAC dose and treatment duration for NE, as well as the relapse risk of skin picking behavior after the cessation of therapy, still require clarification in future studies.

## Financial support

None declared.

## Authors’ contributions

Deren Özcan: The conception and design of the study; acquisition of data; analysis and interpretation of data; drafting the article and revising it critically for important intellectual content; final approval of the version to be submitted.

## Conflicts of interest

None declared.
